# Restoration of keratinocytic phenotypes in autonomous trisomy-rescued cells

**DOI:** 10.1186/s13287-021-02448-w

**Published:** 2021-08-25

**Authors:** Akiko Tanuma-Takahashi, Momoko Inoue, Kazuhiro Kajiwara, Ryo Takagi, Ayumi Yamaguchi, Osamu Samura, Hidenori Akutsu, Haruhiko Sago, Tohru Kiyono, Aikou Okamoto, Akihiro Umezawa

**Affiliations:** 1 Center for Regenerative Medicine, National Center for Child Health and Development Research Institute, 2-10-1 Okura, Setagaya, Tokyo, 157-8535 Japan; 2 Department of Obstetrics and Gynecology, The Jikei University School of Medicine, Tokyo, 105-8471 Japan; 3 Institute of Advanced Biomedical Engineering and Science, Tokyo Women’s Medical University, Tokyo, 162-8666 Japan; 4 Center for Maternal-Fetal, Neonatal and Reproductive Medicine, National Center for Child Health and Development, Tokyo, 157-8535 Japan; 5 Project for Prevention of HPV-related Cancer, Exploratory Oncology Research and Clinical Trial Center, National Cancer Center, Chiba, 277-8577 Japan

**Keywords:** Keratinocytes, Induced pluripotent stem cells, Trisomy 21, Trisomy rescue, 3D skin

## Abstract

**Background:**

An extra copy of chromosome 21 in humans can alter cellular phenotypes as well as immune and metabolic systems. Down syndrome is associated with many health-related problems and age-related disorders including dermatological abnormalities. However, few studies have focused on the impact of trisomy 21 (T21) on epidermal stem cells and progenitor cell dysfunction. Here, we investigated the differences in keratinocytic characteristics between Down syndrome and euploid cells by differentiating cells from trisomy 21-induced pluripotent stem cells (T21-iPSCs) and autonomous rescued disomy 21-iPSCs (D21-iPSCs).

**Methods:**

Our protocol for keratinocytic differentiation of T21-iPSCs and D21-iPSCs was employed. For propagation of T21- and D21-iPSC-derived keratinocytes and cell sheet formation, the culture medium supplemented with Rho kinase inhibitor on mouse feeder cells was introduced as growth rate decreased. Before passaging, selection of a keratinocytic population with differential dispase reactivity was performed. Three-dimensional (3D) air-liquid interface was performed in order to evaluate the ability of iPSC-derived keratinocytes to differentiate and form stratified squamous epithelium.

**Results:**

Trisomy-rescued disomy 21-iPSCs were capable of epidermal differentiation and expressed keratinocytic markers such as *KRT14* and *TP63* upon differentiation compared to trisomy 21-iPSCs. The lifespan of iPSC-derived keratinocytes could successfully be extended on mouse feeder cells in media containing Rho kinase inhibitor, to more than 34 population doublings over a period of 160 days. Dispase-based purification of disomy iPSC-derived keratinocytes contributed epidermal sheet formation. The trisomy-rescued disomy 21-iPSC-derived keratinocytes with an expanded lifespan generated 3D skin in combination with a dermal fibroblast component.

**Conclusions:**

Keratinocytes derived from autonomous trisomy-rescued iPSC have the ability of stratification for manufacturing 3D skin with restoration of keratinocytic functions.

**Supplementary Information:**

The online version contains supplementary material available at 10.1186/s13287-021-02448-w.

## Background

Down syndrome patients have an extra copy of chromosome 21, which causes health-related problems and age-related disorders. Trisomy 21 results in the triplication of over 400 genes, which makes clarification of the precise mechanisms of some phenotypes difficult in Down syndrome [1]. The complications of Down syndrome are numerous; clinical manifestations include mental retardation, congenital heart disease, impaired cognition, premature aging, metabolic problems, and leukemia [2, 3]. Trisomy 21 is also associated with an increased incidence of several dermatological conditions. Immunological disturbances and/or barrier dysfunction of skin are associated with psoriasis, atopic dermatitis, and cutaneous infections. Keratodermatoses and abnormality of elastic fibers also contribute to dermatological disorders such as anetoderma and keratosis pilaris in Down syndrome patients [4–6]. Genetic signatures of these dermatological diseases have been reported using keratinocytes derived from patient-specific iPSCs [7].

A Down syndrome mouse model exhibits hyperproliferation of trisomy 21-keratinocytes in the skin [8]. Cell culture models of trisomy 21 are important for development of conventional therapy for Down syndrome. However, few studies have focused on differences in epidermal characteristics between Down syndrome and euploid cells in vitro. Generation of disease-specific induced pluripotent stem cells (iPSC) allows an alternative paradigm for modeling human genetic disease in vitro [9, 10]. Directed differentiation from these iPSC theoretically enables researchers to derive any given disease-affected human cell-type [11]. Disease-specific iPSCs, therefore, serve as a powerful tool for investigating the mechanisms of Down syndrome [9, 12, 13].

The differences in genetic backgrounds of individuals has hindered the assessment the phenotypic disparities between Down syndrome cells and healthy control cells. Paired disomic/trisomic iPSCs derived from the same trisomic individual by insertion of a transgene into a chromosome 21 has allowed investigation of trisomy 21-dependent gene expression and the impact of an extra copy of chromosome 21 without the confounding effects of a different genetic background [13–16]. The artificial selection of disomic clones possibly results in epigenetic changes that add to the transcriptional differences between the trisomic and disomic iPSCs [17]. In the previous study, we have obtained revertant cells with normal chromosome 21 diploids from the trisomy 21 iPSCs during long-term cultivation [18]. Autonomously rescued disomic cells serve as a good control for trisomic cells. Both disomy 21 iPSC and trisomy 21 iPSC have been shown to differentiate into neural stem cells [18].

In this study, we successfully extended the lifespan of trisomy-rescued iPSC-derived keratinocytes to more than 34 population doublings over a period of 160 days with maintenance of keratinocytic phenotypes. In addition, keratinocytes derived from autonomous trisomy-rescued iPSCs regained capability of stratification and were suitable for manufacturing three-dimensional skin with keratinocytic function.

## Methods

### Cells

iPSCs from Down syndrome patients were cultivated as previously described [19]. Trisomy 21 iPSCs (T21-iPSCs) and rescued disomy 21 iPSCs (D21-iPSCs) were maintained in Essential 8 (E8) medium (Life Technologies, catalog number (#) A1517001) onto vitronectin (VTN) (Life Technologies, #A14700)-coated dishes and passaged using 0.5 mM EDTA in phosphate buffered saline (PBS) [18]. Normal human epidermal keratinocytes (LONZA, #192906) were also cultured in defined keratinocyte serum-free medium (DKSFM) (Invitrogen, #10744-019) supplemented with 10 μM Y-27632 on type I collagen (Advanced Biomatrix, #5005-B) and fibronectin (Sigma-Aldrich, #F0895-1MG)-coated dishes. HDK1-K4DT, which is a normal human keratinocyte line immortalized with TERT, a mutant form of CDK4 and cyclin D1 by lentivirus-mediated gene transfer, was cultured in keratinocyte serum-free medium (Invitrogen, #17005042, low calcium concentration) [20].

### Real-Time qPCR

RNA was extracted from cells using the RNeasy Plus Mini kit (Qiagen, #74134). An aliquot of total RNA was reverse transcribed using an oligo (dT) primer (Invitrogen, #18418-020). For the thermal cycle reactions, the cDNA template was amplified (Applied Biosystems Quantstudio 12K Flex Real-Time PCR System) with gene-specific primer sets (see Additional file 1: Table S1) using the Platinum SYBR Green qPCR SuperMix-UDG with ROX (Invitrogen, #11733-046) under the following reaction conditions: 40 cycles of PCR (95 °C for 15 s and 60 °C for 1 min) after an initial denaturation (95 °C for 2 min). Fluorescence was monitored during every PCR cycle at the annealing step. mRNA levels were normalized using glyceraldehyde-3-phosphate dehydrogenase (GAPDH) as a housekeeping gene. RT-qPCR analyses for expression of epithelial markers were showed as means of percent of GAPDH (%GAPDH) with the standard deviation (SD).

### Immunocytochemical analysis

Cells were fixed with 4% paraformaldehyde in PBS for 10 min at 4 °C. After washing with PBS and treatment with 0.1% Triton X-100 (Sigma-Aldrich, # T8787-100 ML) for 10 min at room temperature, the cells were incubated with Protein Block Serum-Free Ready-to-use (Dako, # X0909) for 30 min at room temperature. This was followed by reaction with primary antibodies in blocking buffer for 60 min at room temperature. After washing with PBS, the cells were incubated with fluorescently conjugated secondary antibodies; anti-rabbit or anti-mouse immunoglobulin G (IgG) bound to Alexa 488 or 546 (1:1,000) was incubated in blocking buffer for 30 min at room temperature. The nuclei were stained with DAPI (Biotium, # 40043). All images were captured using confocal microscopy (LSM 510 and LSM 510 MERA laser scanning microscope; Carl Zeiss, Germany). The fluorescent cells were quantified by hybrid cell counting using a digital microscope (BZ-X 710; Keyence Corp., Osaka, Japan). We measured the number of positive cells in at least six fields to obtain a total of more than 450 cells. Antibody information is provided in Additional file 2: table S2a.

### Immunohistochemical analysis

Immunohistochemistry was performed as previously described [21]. Paraffin sections were deparaffinized and heated in antigen retrieval solution (Nichirei, #415211) for 20 min. After washing with PBS, samples were placed in 3% hydrogen peroxide/methanol for 5 min to block endogenous peroxidase. The sections were then incubated at room temperature for 90 min in primary antibodies diluted with antibody diluent. Antibody information is provided in Additional file 2: table S2b. The sections were then washed three times with PBS and incubated with peroxidase-labeled goat anti-mouse or anti-rabbit antibodies (Nichirei, #424151) at room temperature for 45 min. After washing with PBS, they were incubated in 3,3′-diaminobenzidin (Muto pure chemicals, #40651) for 5–10 min. Negative controls were performed by omitting the primary antibody. The sections were counterstained with hematoxylin.

### Differentiation of iPSCs into keratinocytes

The induction of differentiation into keratinocytes was carried out as previously described [19]. We subcultured small clumps of undifferentiated iPSC on VTN-coated 100-mm dish in E8 medium on day 0. iPSCs were cultured in DKSFM (Invitrogen, #10744-019) supplemented with 1 μM all-*trans* RA (Wako, #182-01111) and 10 ng/mL bone morphogenetic protein 4 (BMP4) (R&D systems, #314-BP-010/CF) from day 1 to day 4. After 4 days, iPSCs were maintained in DKSFM supplemented with 20 ng/mL epidermal growth factor (EGF) (R&D systems, #236-EG-200) for 10 days, then passaged to a 100-mm dish coated with 0.03 mg/mL type I collagen (Advanced Biomatrix, #5005-B) and 0.01 mg/mL fibronectin (Sigma-Aldrich, #F0895-1MG), and maintained in DKSFM supplemented with 20 ng/mL EGF and 10 μM Y-27632 (Wako, #251-00514). Cells were subcultured when approximately 80 to 90% confluence. Cells were rinsed with PBS and incubated with 0.25w/v% trypsin-1mmol/L EDTA (Wako, #209-16941) at 37 °C for 5 min for passaging. Cells were passaged at 0.3 × 10^5^ cells/cm^2^ to a new plate coated with type I collagen and fibronectin and enriched by rapid adherence to fibronectin and type I collagen-coated dished for 15 min at 37 °C. Nonadherent cells were removed and rapidly attached cells were cultured. The medium was changed every 2 or 3 days. All cells were maintained at 37 °C in a humidified incubator with 5% CO_2_.

### Propagation of keratinocytes derived from iPSCs

For proliferation of iPSC-derived keratinocytes, we employed specific culture conditions suitable for keratinocytes [22–24]. iPSC-derived keratinocytes were cultured in ESTEM-EP medium (GlycoTechnica Ltd., Japan) [Dulbecco’s modified Eagle’s medium (DMEM) +F12 (3:1) medium supplemented with FBS, hydrocortisone, adenine, EGF, cholera toxin, insulin, A83-01, nicotinamide, SB202190, Y-27632, and penicillin/streptomycin] onto irradiated mouse embryo fibroblasts (MEF). The medium was changed every 2 or 3 days until the cells were reached 80 to 90% confluence. Cells were passaged at 0.1–0.25 × 10^5^ cells/cm^2^ to a new plate of irradiated MEF feeder cells. The cell sheets were retrieved from the dish with 10 mg/mL dispase (Wako, #383-02281) or harvested using a cell scraper. The sheets in iPGell (Genostaff, #PG20-1) were prepared for immunohistochemistry. The cell numbers were recorded at each passage using the Countess automated cell counter and population doublings were determined.

### Dispase-based keratinocytic selection

Before passaging, selection of a keratinocytic population with differential dispase reactivity was performed. Co-culture of iPSC-derived keratinocytes and MEF under culture condition B were rinsed with PBS and incubated with 10 mg/mL dispase at 37 °C for 5–8 min, with close monitoring by phase microscopy. When non-keratinocytes began to detach by tapping the dish, dispase was inactivated, and the solution containing non-keratinocytes and feeder cells were removed by aspiration. Stratified colonies remained tightly adherent. The remaining colonies were rinsed with PBS and incubated with trypsin-EDTA solution at 37 °C for about 3 min. The cells were passaged to a new plate of irradiated MEF feeder cells at a density of 0.1–0.25 × 10^5^ cells/cm^2^.

### Generation of a 3D skin equivalent

Three-dimensional skin was generated according to a previously described protocol [25]. Type I collagen (Koken, #IPC-50) and 1 × 10^6^ human foreskin fibroblasts (HFFs) were mixed and poured into an untreated 60-mm Petri dish (Falcon, #351007) while cooling and allowed to gel at 37 °C for 1 h to prepare the dermal equivalent. The collagen gel was detached from the surface of the Petri dish, and the contraction of the gel was facilitated. The medium was changed every 2 or 3 days for 7 days. iPSC-derived keratinocytes were plated at 0.2 × 10^6^ cells inside in a glass ring (IWAKI, #RING-12) on the surface of the contracted collagen gel, which was plated onto polyethylene terephthalate membranes (Corning, #35-3493). iPSC-derived keratinocytes were grown in ESTEM-EP medium for 1 day, following which they were exposed to air in a 1:1 mixture of ESTEM-EP medium and DMEM supplemented with 10% FBS (medium for HFFs), in which the Ca^2+^ concentration was adjusted to 0.9 mM. The medium was changed every 2 or 3 days. Multilayered 3D cultures of keratinocytes were obtained by day 14.

### Statistical analysis

The results are expressed as means with SD. Comparisons between the two groups were evaluated with the unpaired Student’s t test. For three groups, a one-way analysis of variance (ANOVA) with a Bonferroni correction was used. Statistical analysis was performed using Stata 14.0 (StataCorp LP, College Station, Texas, USA). P values less than 0.05 were considered to be statistically significant.

## Results

### Rescued disomy 21 iPSCs, but not trisomy 21 iPSCs, are likely to differentiate into keratinocytes

**Fig. 1:**
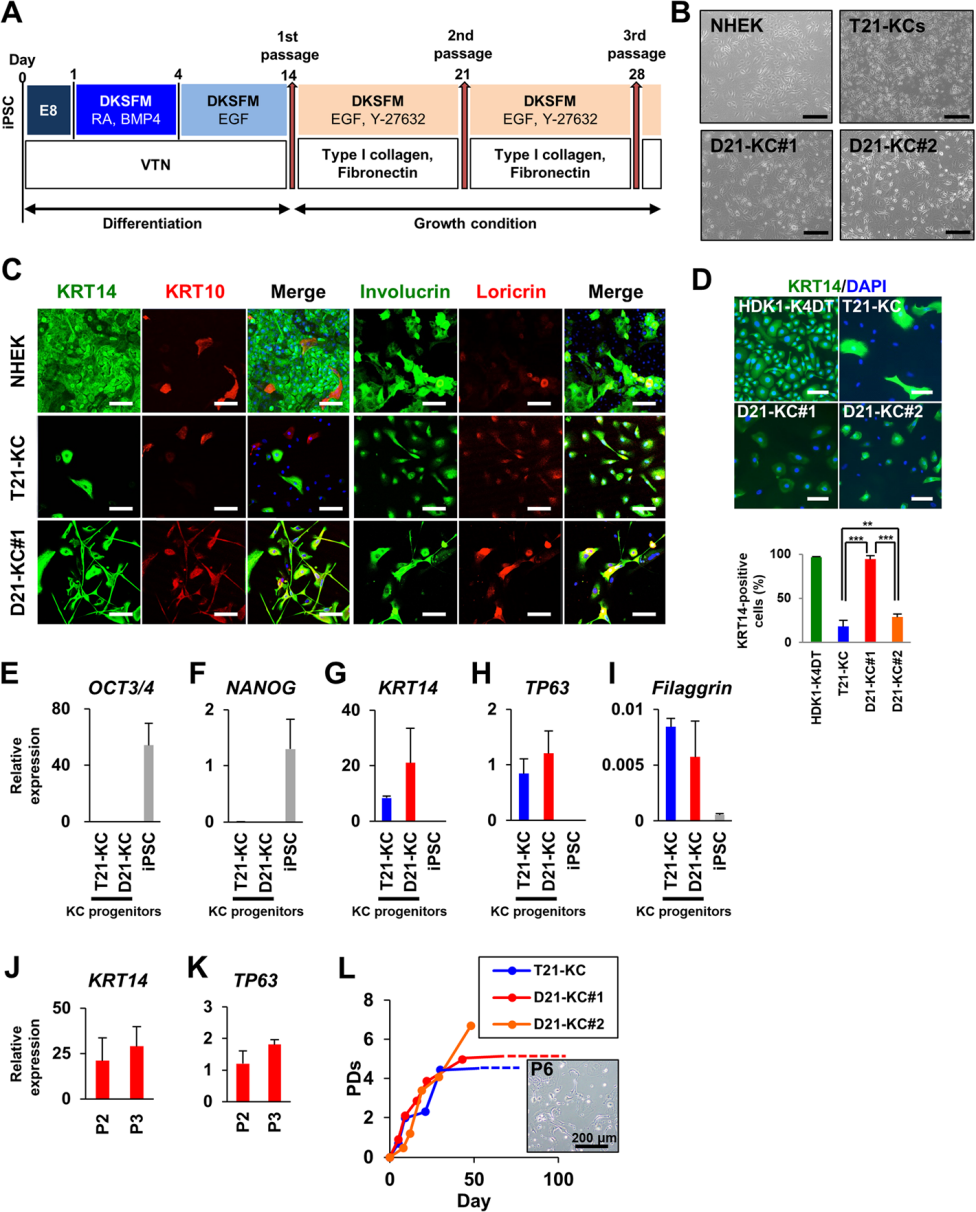
Keratinocytic differentiation of Down syndrome-iPSCs and autonomously rescued disomy 21-iPSCs. **a** Protocol for keratinocytic differentiation from amniotic fluid-iPSCs. DKSFM, defined keratinocyte serum-free medium; RA, retinoic acid; BMP4, bone morphogenetic protein 4; VTN, vitronectin; E8, Essential 8 medium; EGF, epidermal growth factor. **b** Phase-contrast photomicrograph of normal human epidermal keratinocytes (NHEK) at passage 3, and keratinocytes derived from T21-iPSCs (T21-KC), D21-iPSC#1 (D21-KC#1) and D21-iPSC#2 (D21-KC#2) at passage 2 (day 25). T21-KC, D21-KC#1, and D21-KC#2 exhibited keratinocyte-like morphology. Scale bars, 200 μm. **c** Immunocytochemical analysis of T21-KC at day 28 and D21-KC#1 at day 34 with the antibodies to epithelial markers, i.e., KRT14, KRT10, involucrin and loricrin. Scale bars, 100 μm. **d** Immunocytochemistry with the epithelial stem cell marker KRT14. The percentage of KRT-positive cells is shown for HDK1-K4DT (control), T21-KCs at day 56, D21-KC#1 at day 55, and D21-KC#2 at day 57. Scale bars, 100 μm. **p < 0.01, ***p < 0.001, one-way ANOVA with Bonferroni correction. **e** Real-time qPCR analysis of *OCT3/4* in T21-KCs at day 28, D21-KC#1 at day 28, and undifferentiated iPSCs. Values are shown as means ± SD from three independent experiments. **f** Real-time qPCR analysis of *NANOG* in T21-KC at day 28, D21-KC#1 at day 28, and undifferentiated iPSCs. Values are shown as means ± SD from two or three independent experiments. **g** Real-time qPCR analysis of *KRT14* in T21-KC at day 28, D21-KC#1 at day 28, and undifferentiated iPSCs. The expression level of *KRT14* was relatively high in D21-KCs, compared with T21-KCs (p-value, 0.42). Values are shown as means ± SD from three independent experiments. **h** Real-time qPCR analysis of *TP63* in T21-KC at day 28, D21-KC#1 at day 28, and undifferentiated iPSCs. The expression level of *TP63* was relatively high in D21-KCs, compared with T21-KCs (p-value, 0.31). Values are shown as means ± SD from three independent experiments. **i** Real-time qPCR analysis of *Filaggrin* in T21-KC at day 28, D21-KC#1 at day 28, and undifferentiated iPSCs. The expression level of *Filaggrin* was relatively high in T21-KCs, compared with D21-KCs (p-value, 0.34). Values are shown as means ± SD from three independent experiments. **j** Real-time qPCR analysis of *KRT14* in D21-KC#1 at passages 2 and 3. Values are shown as means ± SD from three independent experiments. **k** Real-time qPCR analysis of *TP63* in D21-KC#1 at passages 2 and 3. Values are shown as means ± SD from three independent experiments. **l** Growth of iPSC-derived keratinocytes. The total number of population doublings (PDs) was calculated using the formula [log10 (total number of harvested cells/number of plated cells)]/log10 (2). Phase-contrast photomicrograph of D21-KC#1 at senescence is shown

We employed a standard protocol for differentiation of T21-iPSCs and D21-iPSCs into keratinocytes (Fig. 1a). The differentiated cells derived from T21- and D21-iPSCs, i.e., keratinocyte progenitor cells, resembled normal human epidermal keratinocytes (NHEK) at passage 2 (Fig. 1b). Gene expression analysis was performed using iPSC-derived keratinocytes at passage 2 and/or passage 3. Immunostaining analysis revealed that both keratinocytes derived from T21-iPSC (T21-KC) and D21-iPSC#1 (D21-KC#1) were positive for epithelial markers, i.e., KERATIN 14 (KRT14), KRT10, involucrin, and loricrin (Fig. 1c). T21-KCs at passage 5 had a significantly low number of KRT14-positive cells, compared to HDK1-K4DT and D21-KC at passage 5 (Fig. 1d). The number of Involucrin-positive cells in both T21- and D21-KCs was low, compared with NHEK (Fig. 1c). T21-KCs also contained non-keratinocytes such as iPSC-like colonies and cells and exhibited loss of nuclear-cytoplasmic boundaries at passage 3 (see Additional file 3: Fig. S1A). It was extremely difficult to maintain T21-KCs with keratinocyte morphology. In contrast, D21-KC#1 and #2 showed keratinocytic morphology and KRT14 reactivity in most cells, like NHEK cells (Fig. 1c; Additional file 3: Fig. S1B). The expression of the *OCT3/4* and *NANOG* genes was significantly suppressed after differentiation of T21-iPSCs and D21-iPSCs (Fig. 1e, f). The basal cell marker *KRT14*, the epithelial progenitor marker *TP63*, and the keratinizing cell marker *Filaggrin* were induced in T21-KC and D21-KC#1 (Fig. 1g–i). The expression of *KRT14* and *TP63* was relatively increased in D21-KCs, compared with T21-KCs (Fig. 1g, h). *Filaggrin* expression was relatively high in T21-KCs (Fig. 1i). The expression of *KRT14* and *TP63* were unchanged in D21-KC#1(Fig. j, k). T21-KC, D21-KC#1, and D21-KC#2 had keratinocytic morphology stopped dividing at 4 to 5 population doublings (PDs) (Fig. 1l). These results imply that T21- and D21-iPSCs have a distinct propensity for keratinocytic differentiation.

### A combination of serum, Y-27632, and feeder cells allows propagation and sheet formation of D21-KCs

**Fig. 2:**
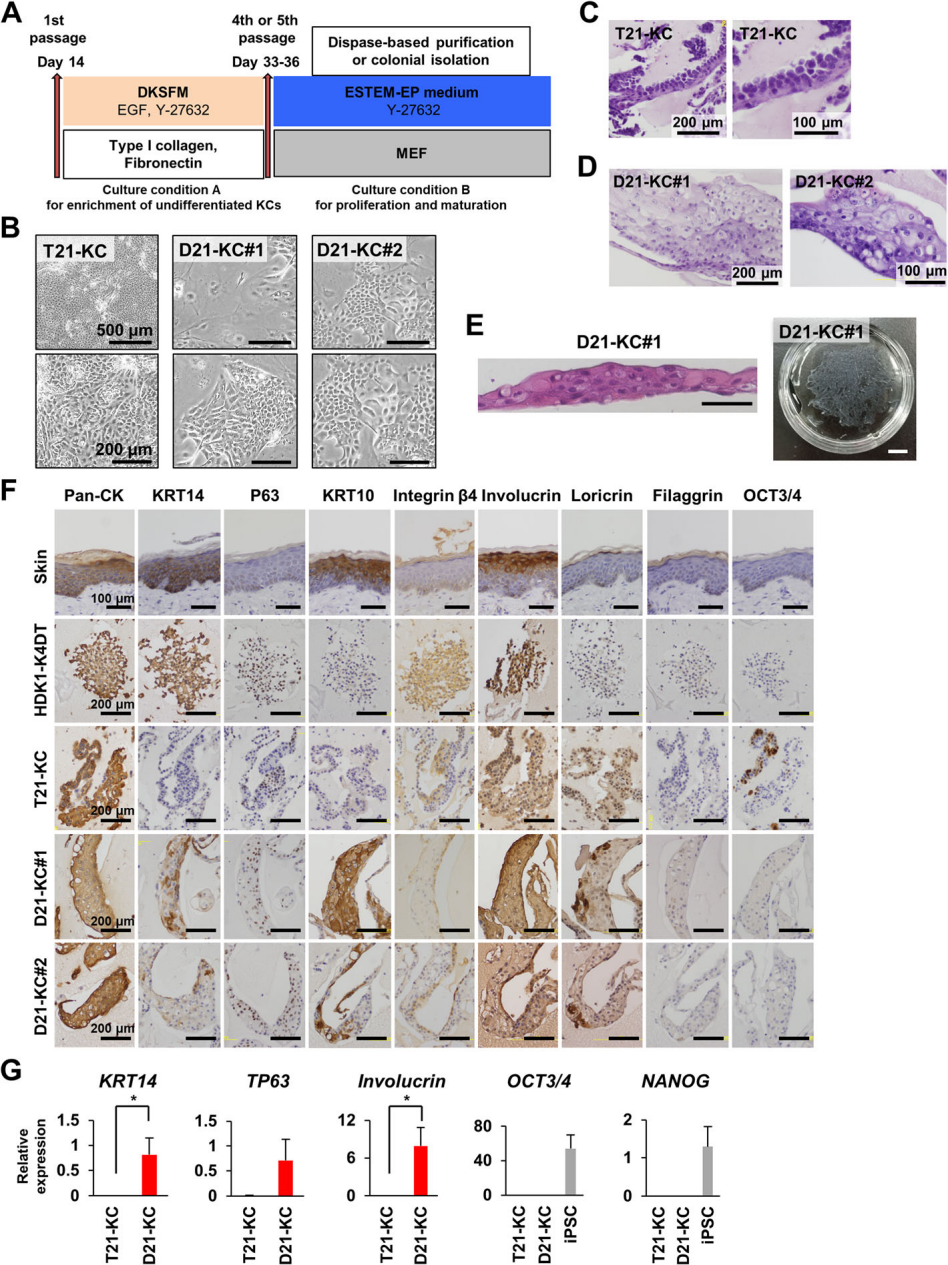
Sheet formation and characterization of keratinocytes derived from T21- and D21-iPSC. **a** Schematic diagram of the experimental design. Keratinocytes derived from iPSCs were cultivated under culture condition A followed by condition B for the purpose of propagation of the lifespan. As the growth rate decreased, the culture conditions were changed from A to B. In the culture condition A, keratinocytes were maintained in DKSFM supplemented with 20 ng/mL EGF and 10 μM Y-27632 at a plate coated with type I collagen and fibronectin. In culture condition B, keratinocytes were cultured in the ESTEM-EP medium supplemented with 10 μM Y-27632 on irradiated mouse embryo fibroblasts (MEF). **b** Phase-contrast photomicrographs of keratinocytes derived from T21-KC at passage 4, D21-KC#1 at passage 5, and D21-KC#2 at passage 4. **c** Thin section of T21-KC at day 42 in iPGell. Hematoxylin and eosin (HE) stain. **d** Thin section of D21-KC#1 at day 50 and D21-KC#2 at day 48 in iPGell. HE stain. **e** Fabrication of D21-KC#1 epithelial sheet after dispase treatment. (Left panel) Histology of the sheet. HE stain. Scale bar, 100 μm. (Right panel) Macroscopic view of the sheet. Scale bar, 1 cm. **f** Immunohistochemical analysis of skin, HDK1-K4DT, T21-KCs at day 42, D21-KC#1 at day 50, and D21-KC#2 at day 48 with antibodies to pan-cytokeratins (Pan-CK), KRT14, P63, KRT10, integrin β4, involucrin, loricrin, filaggrin, and OCT3/4. D21-KC exhibited terminal differentiation and formed structured cell sheets. The expression patterns of these markers in intact skin are shown for reference. **g** Real-time qPCR analysis of epithelial markers (*KRT14, TP63, Involucrin*) and pluripotent markers (*OCT3/4, NANOG*) in T21-KC at day 47, D21-KC#1 at day 49, and undifferentiated iPSCs. Values are shown as means ± SD from two or three independent experiments. *p < 0.05

To escape cell senescence and form epidermal sheets, we altered the cultivation conditions (Fig. 2a). T21-KCs, D21-KC#1, and D21-KC#2 recovered the ability to grow and proliferated as colonies on MEFs (Fig. 2b). The differentiated cells under these culture conditions were defined as mature keratinocytes. We analyzed these cells 1–2 weeks after the change in cultivation conditions. T21-KCs showed multilayer structures with a high nucleus/cytoplasm (N/C) ratio and chromatin aggregation (Fig. 2c). In contrast, D21-KC#1 and D21-KC#2 cells showed an epithelial structure with basal, spinous, and granular layers (Fig. 2d). Uniformly thick multilayer-sheets were obtained by dispase treatment (Fig. 2e). Immunostaining of D21-KC stratified epithelium revealed layer-dependent reactivity to KRT14, basement membrane integrin β4, the suprabasal marker KRT10, and spinous and granular layer markers involucrin (Fig. 2f). Loricrin, a major component of granular layers, was also detected in the KRT14-negative cells. In contrast to D21-KC#1 and D21-KC#2 cells, T21-KC expressed OCT3/4 in some cells and failed to express KRT10 and KRT14. These results suggest that the autonomously rescued disomy 21 keratinocytes produced epithelial structures. Along with the immunocytochemical analysis, RT-qPCR analysis revealed a lack of expression of epithelial markers *KRT14, TP63,* and *Involucrin* in T21-KCs (Fig. 2g). D21-KC#1, but not T21-KCs, significantly increased in the epithelial markers after the change in culture conditions. These significant increases in keratinocytic marker expression are probably due to culture condition B, which is suitable for keratinocyte proliferation and maturation (Fig. 2a). The expression of *OCT3/4* and *NANOG* diminished in both T21- and D21-KCs. Cytological analysis showed that T21-KCs, D21-KC#1, and D21-KC#2 treated with condition A showed variable cell sizes with a low N/C ratios (see Additional file 4: Fig. S2).

### Trisomy 21- and rescued disomy 21-iPSCs escape from senescence for more than 160 days

**Fig. 3:**
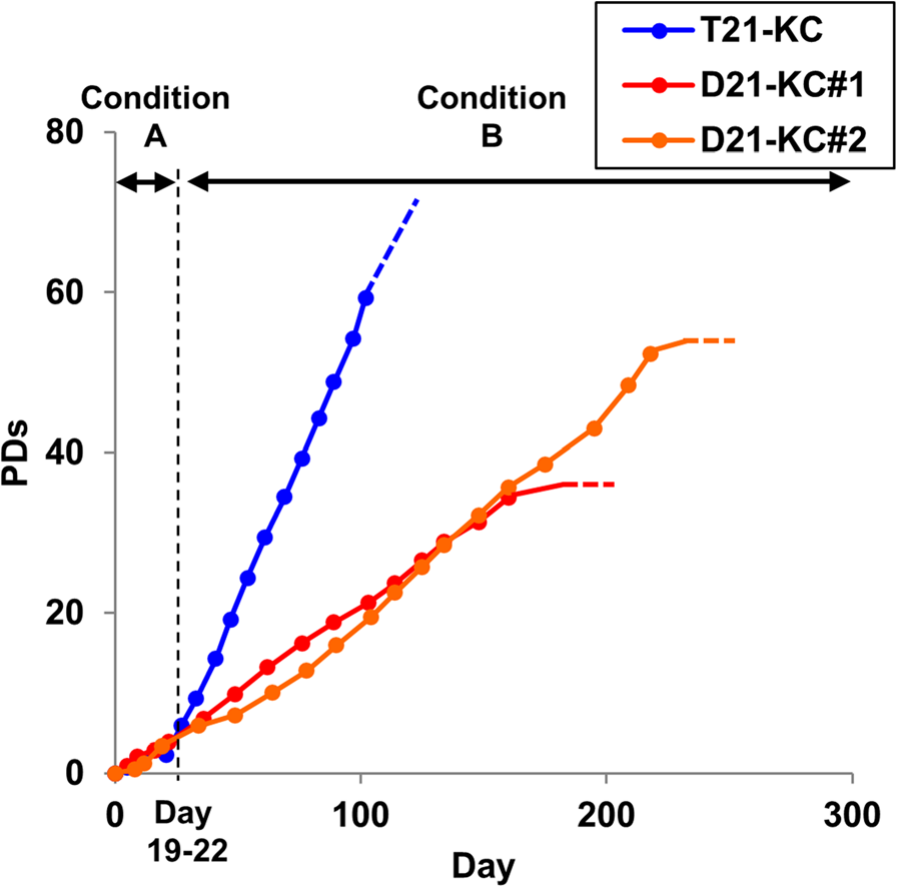
Differentiating cells from T21-iPSCs and the rescued disomy (D21)-iPSCs shows proliferative capability for more than 160 days. The numbers of cells were recorded at each passage using an automated cell counter and population doublings were determined. The total number of population doublings (PDs) was calculated using the formula [log10 (total number of harvested cells/number of plated cells)]/log10 (2) from two or three independent experiments

The proliferative capability of T21-KC, D21-KC#1, and D21-KC#2 was restored after the change in cultivation conditions (Fig. 3). T21-KC cells continued to proliferate rapidly and had fewer cells that showed keratinocytic morphology. In contrast, D21-KC#1 and D21-KC#2 exhibited stable growth and maintained keratinocytic morphology for a period of more than 160 days. D21-KC#1 and D21-KC#2 showed growth cessation at 34 and 52 PDs, respectively. The reason for the differences in growth cessation is unclear but may be related to the differentiation competency of these cells.

### Dispase-based purification of iPSC-derived keratinocytes improves epidermal sheet formation

**Fig. 4:**
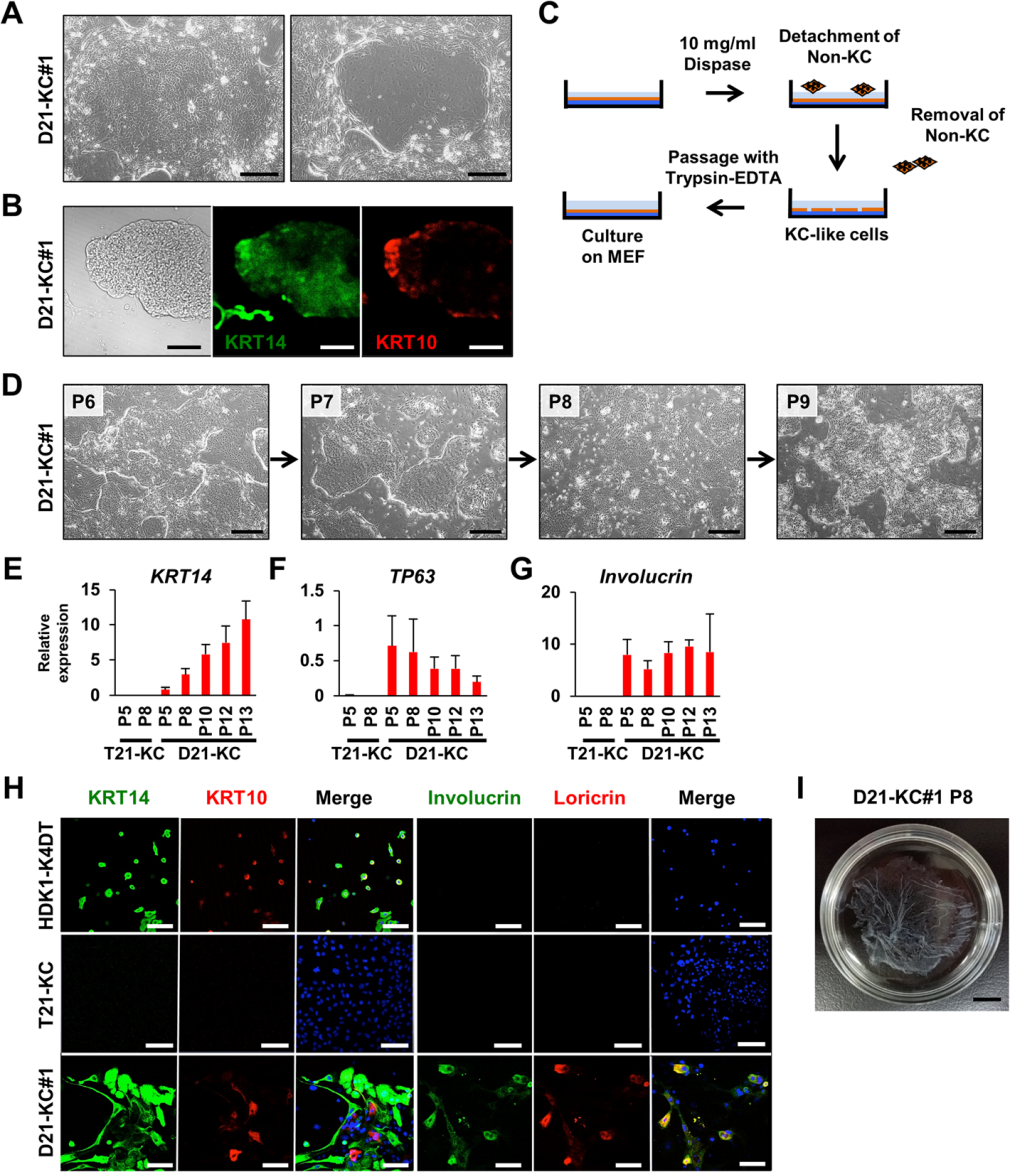
Purification of iPSC-derived keratinocytes, based on differential reactions to dispase. **a** Phase-contrast photomicrographs of keratinocytes derived from D21-KC#1 at passage 5. Two types of colonies could be observed: stratified keratinocyte colonies (left) and non-keratinocyte-like cell colonies (right). Scale bars, 200 μm. **b** Immunocytochemical analysis of D21-KC#1 colony with epithelial markers, i.e., KRT14 and KRT10. Scale bars, 200 μm. **c** Selection of keratinocytes with dispase digestion. Non-keratinocytes (non-KC) detached earlier than keratinocytes with dispase treatment. Non-keratinocytes and feeder cells were removed at each passage. Keratinocytes were then passaged. **d** Phase-contrast photomicrographs of D21-KC#1 during the dispase selection. There were insular colonies, which were formed by stratified epithelial cells, and another area contained non-keratinocyte-like cells. Dispase-based selection at each passage caused non-keratinocyte-like cells to decrease. Scale bars, 500 μm. **e** Real-time qPCR analysis of *KRT14* at each passage. Values are shown as means ± SD from two or three independent experiments. **f** Real-time qPCR analysis of *TP63* at each passage. Values are shown as means ± SD from two or three independent experiments.** g** Real-time qPCR analysis of *Involucrin* at each passage. Values are shown as means ± SD from two or three independent experiments. **h** Immunocytochemical analysis of epithelial markers, i.e., KRT14, KRT10, involucrin, and loricrin after colony isolations (T21-KC) at passage 7 and dispase-based selection (D21-KC#1) at passage 13. Scale bars, 100 μm. **i** Epithelial sheets of keratinocyte-like cells derived from D21-iPSC#1 at passage 8. Cell sheets were harvested by dispase treatment. Scale bar, 1 cm

After long-term cultivation, D21-KC#1 cell cultures contained both keratinocytes and non-keratinocytes (Fig. 4a). D21-KC#1 keratinocyte-like cells expressed KRT14 in most cells of the stratified colonies and KRT10 at the periphery of the colonies (Fig. 4b). To separate keratinocyte-like cells from non-keratinocyte-like cells, we used differential reactivity of these cells to dispase, i.e., tight or loose cell-dish adhesion: Stratified colonies are relatively resistant to dispase and non-keratinocytes are easily detached after dispase treatment (Fig. 4c). This treatment was applied to D21-KC#1 cells at passage 7 to passage 9 (Fig. 4d). The ratio of keratinocytes increased at each passage by microscopic observation with a phase-contrast microscope. *KRT14* increased, *TP63* decreased, and *Involucrin* remained unchanged at each passage (Fig. 4e–g). D21-KC#1 was immunocytochemically positive for epidermal markers, i.e., KRT14, KRT10, involucrin, and loricrin, at passage 13 (Fig. 4h). D21-KCs were able to reproducibly generate epidermal sheets (Fig. 4i). T21-KCs became fragmented upon dispase treatment; thus, we decided not to use dispase for keratinocytic purification. We picked up colonies with keratinocyte morphology at each passage from passage 6 to passage 8 (see Additional file 5: Fig. S3A, B). Keratinocyte-like cells were microscopically observed at passage 8; however, these cells did not express *KRT14* and *TP63* and epithelial markers were absent (Fig. 4e, f, h). The lack of keratinocyte marker expression may be caused by a gene dosage imbalance due to trisomy 21.

### 3D skin can be manufactured from disomy 21 iPSC-derived keratinocytes

**Fig. 5:**
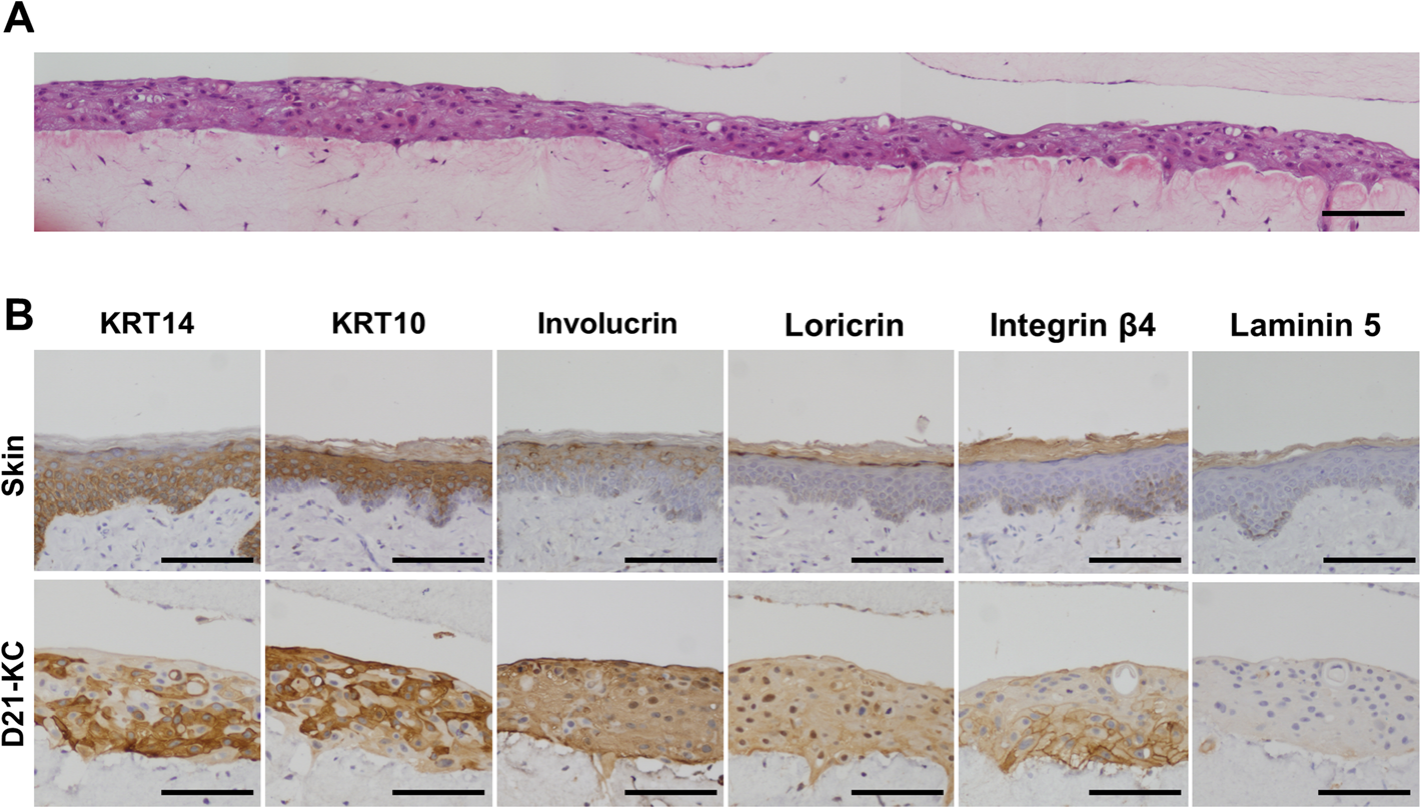
Three-dimensional (3D) cultured skin equivalents from D21-KC#1. **a** Histology of the 3D keratinocytes from D21-iPSCs. HE stain. Scale bar, 200 μm. **b** Immunohistochemical analysis of the expression and location of epithelial markers, i.e., KRT14, KRT10, involucrin, loricrin, integrin β4, and laminin 5. The expression patterns of these markers in intact skin are shown for reference. Scale bars, 200 μm

The dispase-based selection at each passage helped maintain the proliferation of KRT14 positive cells and stratified cell sheets were manufactured in plate cultures of D21-KC#1. We therefore investigated whether disomy 21 iPSC-derived keratinocytes could manufacture 3D skin. To this end, we generated artificial dermis by combining human cultured dermal cells and type I collagen in a Petri dish, and overlaid D21-KC#1 cells in a glass ring. D21-KC#1 cells on the artificial dermis were then cultivated in an air-liquid interface for 2 weeks to accelerate epidermal differentiation. The 3D skin from D21-KC#1 was generated with irregular layer formation and expressed KRT14, KRT10, involucrin, loricrin, and integrin β4 (Fig. 5a, b). Laminin 5 was not detected at the dermal-epidermal junction.

## Discussion

### Differentiation incompetence in T21-iPSCs and its restoration in D21-iPSCs

Restoration of differentiation and growth of trisomy-rescued iPSCs is a focus of our laboratory. This restoration or normalization may simply be attributable to a gene dosage effect, i.e., a change from 3 (aneuploid) to 2 copies (euploid). The Down syndrome phenotype is generally derived from the increased expression levels of dosage-sensitive genes, and most of the triplicated genes show upregulated expression that is compatible with gene dosage (i.e., at close to 1.5-fold increase) [15, 26]. The expression levels of the genes for APP (Alzheimer’s disease marker), DYRK1A, DSCR1 (Down syndrome critical region 1), ETS2, and SOD1, all of which are located on chromosome 21, are dysregulated in trisomic cells. Although a gene dosage imbalance is the main molecular mechanism, extensive dysregulation of euploid genes are associated with Down syndrome phenotypes [26, 27]. An extra copy of chromosome 21 also alters gene expression across every chromosome, not just chromosome 21 [9, 28]. The altered expression levels decrease to normal levels in trisomy-rescued undifferentiated iPSCs; in other words, revertant cells regain the gene expression levels of intact iPSCs [18]. These normalized gene expression levels may restore keratinocytic differentiation capability in trisomy-rescued iPSCs. The detailed mechanism of keratinocytic restoration in trisomy 21 cells remains unclear, and additional analysis may elucidate the mechanism underlying epidermal abnormalities in Down syndrome.

The ability of trisomy 21-stem/progenitor cells to differentiate also depends on the target organs or cells [14, 15, 29, 30]. The downregulated genes in T21-iPSCs reveal significant enrichment for genes involved in embryonic and tissue morphogenesis [15]. In addition, differentiated ectoderm germ structures are not found in T21-iPSC-derived teratomas [31]. In another study, lack of pluripotency in T21-iPSCs with reduced expression of HERVH might explain the lack of T21-iPSC differentiation compared to disomic iPSCs [15]. These reports may explain the inability of to T21-iPSCs differentiate, i.e., a lack of KRT14 marker and the partial expression of OCT3/4 in T21-KCs, and increased population of non-keratinocytes after the long-term culture. It is noted that keratinocytic differentiation of D21-iPSCs was achieved at passage 106. This implies that the differentiation capability of trisomy-rescued cells is extremely stable for a period of more than 500 days.

### Uncontrolled growth of T21-KCs and normalized growth of D21-KCs

Accelerated growth of differentiated cells from T21-iPSCs into keratinocytes was unexpected because the incidence of solid tumors, such as dermatological tumors, is decreased in Down syndrome patients [32, 33]. Candidate genes such as *DYRK1A, PIGP,* and *RCAN1* are related to keratinocyte hyperproliferation in a Down syndrome mouse model [8]. Triplication of *Usp16*, located on chromosome 21, reduces self-renewal of hematopoietic stem cells and expansion of mammary epithelial cells, neural progenitors, and fibroblasts [34]. Overexpression of *patched-1*, a receptor that represses the mitogenic sonic hedgehog pathway, may be associated with proliferation impairment in skin [35–38]. Moreover, proliferation of trisomy 21-fibroblasts and trisomy 21-iPSCs decrease due to oxidative stress and protein aggregation [39, 40]. Neurogenesis-related genes such as *DYRK1A* and *RCAN1*, craniofacial defect-related genes such as *DYRK1A, ETS2,* and *RCAN1*, and tumor suppressor-related genes such as *DYRK1A, ETS2,* and *RCAN1* were also reported in Down syndrome [26]. In our study, an increased growth rate, an increased nucleus/cytoplasm ratio, and aggregated chromatin of T21-KCs may have resulted from susceptibility to growth stimulation. Skin tumor growth and hyperkeratosis were detected in Ts1Rhr mice model of Down syndrome [8]. We have also reported accelerated growth of T21-derived neural stem cells [18]. These increases in growth of other cell types are indeed compatible with that of T21-KCs in this study.

Psoriasis is one of the dermatological abnormalities observed in Down syndrome and is characterized by hyperproliferation and defective differentiation of keratinocytes. Altered gene expression such as upregulation of IFN genes is linked to hyperproliferation of KCs derived from psoriasis patient-specific iPSCs [7]. The autoimmune abnormalities and altered sensitivity to stimuli in Down syndrome may be linked to the excessive proliferation of T21-KCs and reduced differentiation of T21-iPSCs.

In addition to growth rate, senescence or aging of iPSC-derived keratinocytes is a concern. Human primary keratinocytes in serum-free and chemically defined media senesce around 15-20 PDs [41, 42]. iPSC-derived keratinocytes exhibit growth arrest when cultured in feeder-free, serum-free medium containing EGF and Y-27632 around passage 4 or 5 [19]. Combination of Rho kinase inhibitor and feeder cells induces conditional reprograming and immortalization of human epithelial cells without the use of viral infection or genetic modification [22–24]. In line with these reports, D21-KC survival and proliferation was achieved with a combination of a Rho kinase inhibitor and feeder cells. The successful extension of the D21-KC lifespan to more than 34 population doublings over the period of 160 days is surprising, since extended lifespan of T21-KCs was not accompanied by keratinocytic phenotypes. The trisomy-rescued D21-KCs with a longer lifespan have the ability to stratify, which is essential for manufacturing 3D skin with keratinocytic functions.

## Conclusion

To study the impact of trisomy 21 on keratinocytic function, we provide an iPSC-derived model using trisomy 21 iPSC and autonomous trisomy-rescued iPSCs. Our results suggest that there is impairment in keratinocytic differentiation in trisomy 21 iPSCs. In contrast, propagation of trisomy-rescued iPSC-derived keratinocytes with purification and manufacturing of stratified epithelial sheets and 3D skin imply a restoration of keratinocytic functions. Further investigation of the influence of the extra copy of chromosome 21 may help determine the underlying causes of Down syndrome phenotypes and lead to the generation of a Down syndrome model of dermatological abnormalities.

## Supplementary Information

**Additional file 1: Table S1.** List of primers

**Additional file 2: Table S2.** List of antibodies for immunochemistry

**Additional file 3: Figure S1.** Phase-contrast photomicrographs of T21-KC and D21-KC#1 at passage 3 (Related to Fig. 1). **A**. Phase-contrast photomicrographs of T21-KC cells at passage 3. Left panel: iPSC-like colony, middle panel: non-epithelial cells, right panel: cells with loss of nuclear-cytoplasmic boundary. **B**. Phase-contrast photomicrographs of D21-KC#1 cells at passage 3. Left and middle panels: keratinocyte-like cells, right panel: cells with loss of nuclear-cytoplasmic boundary.

**Additional file 4: Figure S2.** Characterization of keratinocyte derived from iPSCs (Related to Fig. 2). Left panels: Phase-contrast photomicrographs of HDK1-K4DT (normal human keratinocytes) at passage 14, T21-KC at passage 4, D21-KC#1 at passage 5, and D21-KC#2 at passage 4 in a defined keratinocyte serum-free medium (DKSFM), i.e., culture condition A. Right panels: Thin sections of T21-KC at passage 4, D21-KC#1 at passage 5, and D21-KC#2 at passage 4. These cells did not adhere each other because of low-calcium medium (DKSFM). HE stain.

**Additional file 5 Figure S3.** Colonial isolation of T21-KC (Related to Fig. 4). **A** Phase-contrast photomicrographs of T21-KC before colony isolation. **B** Phase-contrast photomicrographs of T21-KC after three colony isolations. After three times of isolations from passage 6 to passage 8, keratinocyte-like-cells were observed all over the dish. However, Fig. 4H showed that cytokeratins were not expressed in these cells.

## Data Availability

The datasets used and/or analyzed during the current study are available from the corresponding author on reasonable request.

## Abbreviations

iPSCs: Induced pluripotent stem cells
T21-iPSCs: Trisomy 21-induced pluripotent stem cells
D21-iPSCs: Autonomous rescued disomy 21-iPSCs
3D: Three-dimensional
E8: Essential 8
VTN: Vitronectin
DKSFM: Defined keratinocyte serum-free medium
GAPDH: Glyceraldehyde-3-phosphate dehydrogenase
SD: Standard deviation
BMP4: Bone morphogenetic protein 4
EGF: Epidermal growth factor
DMEM: Dulbecco’s modified Eagle’s medium
MEF: Mouse embryo fibroblasts
HFFs: Human foreskin fibroblasts
NHEK: Normal human epidermal keratinocytes
KRT: KERATIN
KC: Keratinocytes
T21-KC: Keratinocytes derived from T21-iPSC
D21-KC: Keratinocytes derived from D21-iPSC
N/C: Nucleus/cytoplasm
PDs: Population doublings
HE: Hematoxylin and eosin
