# Supplementary figures and images for: Restoration of keratinocytic phenotypes in autonomous trisomy-rescued cells
Source: Stem Cell Res Ther. 2021 Aug 25;12:476. doi: 10.1186/s13287-021-02448-w (PMC8390253; doi:10.1186/s13287-021-02448-w)

## Slide 1
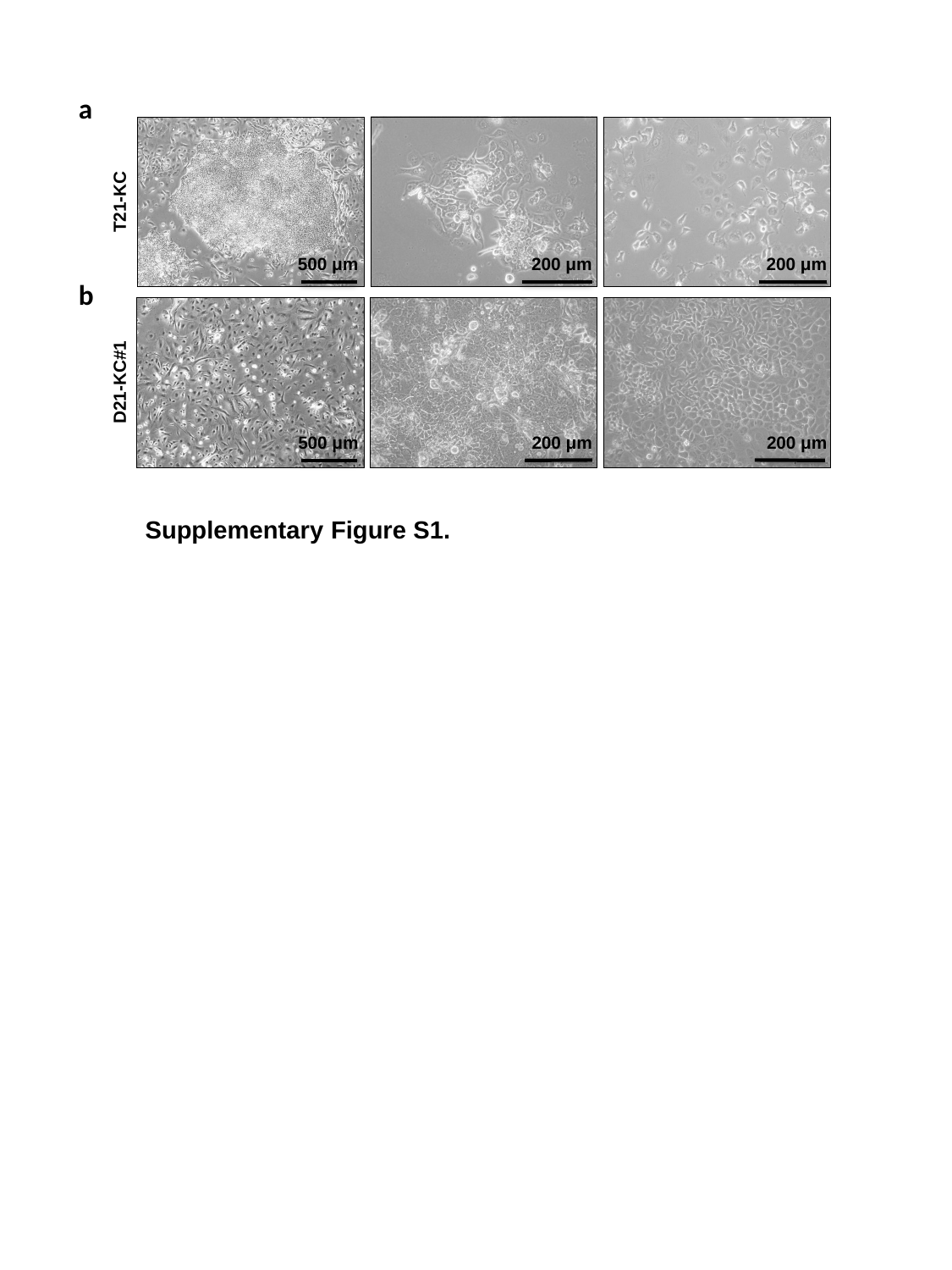

a
T21-KC
500 μm
200 μm
200 μm
b
D21-KC#1
500 μm
200 μm
200 μm
Supplementary Figure S1.

Supplement: Supplementary file 3 — Additional file 3: Figure S1. Phase-contrast photomicrographs of T21-KC and D21-KC#1 at passage 3 (Related to Fig. 1). A. Phase-contrast photomicrographs of T21-KC cells at passage 3. Left panel: iPSC-like colony, middle panel: non-epithelial cells, right panel: cells with loss of nuclear-cytoplasmic boundary. B. Phase-contrast photomicrographs of D21-KC#1 cells at passage 3. Left and middle panels: keratinocyte-like cells, right panel: cells with loss of nuclear-cytoplasmic boundary. [file 13287_2021_2448_MOESM3_ESM.pptx]

## Slide 1
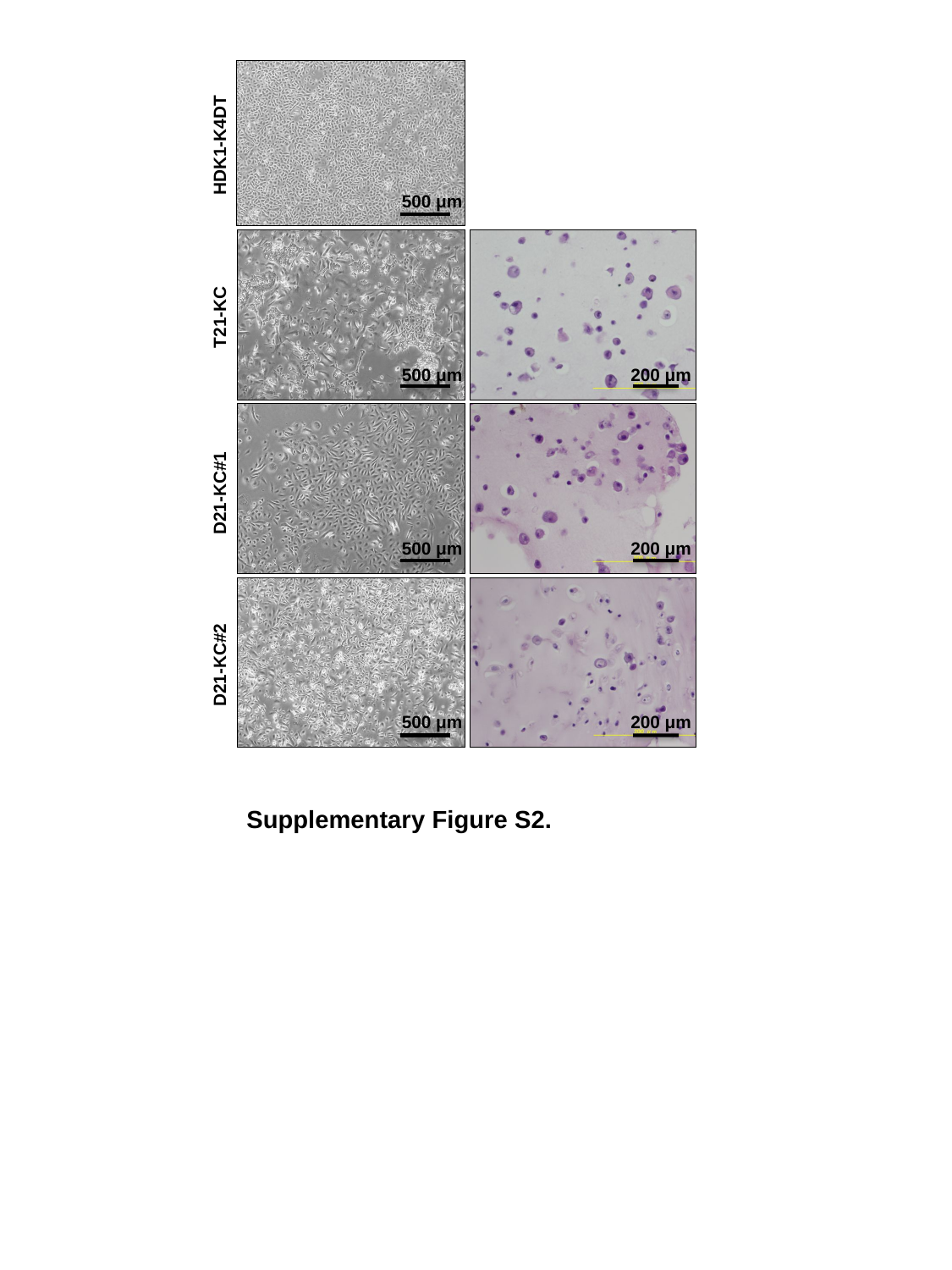

HDK1-K4DT
500 μm
T21-KC
500 μm
200 μm
D21-KC#1
500 μm
200 μm
D21-KC#2
500 μm
200 μm
Supplementary Figure S2.

Supplement: Supplementary file 4 — Additional file 4: Figure S2. Characterization of keratinocyte derived from iPSCs (Related to Fig. 2). Left panels: Phase-contrast photomicrographs of HDK1-K4DT (normal human keratinocytes) at passage 14, T21-KC at passage 4, D21-KC#1 at passage 5, and D21-KC#2 at passage 4 in a defined keratinocyte serum-free medium (DKSFM), i.e., culture condition A. Right panels: Thin sections of T21-KC at passage 4, D21-KC#1 at passage 5, and D21-KC#2 at passage 4. These cells did not adhere each other because of low-calcium medium (DKSFM). HE stain. [file 13287_2021_2448_MOESM4_ESM.pptx]

## Slide 1
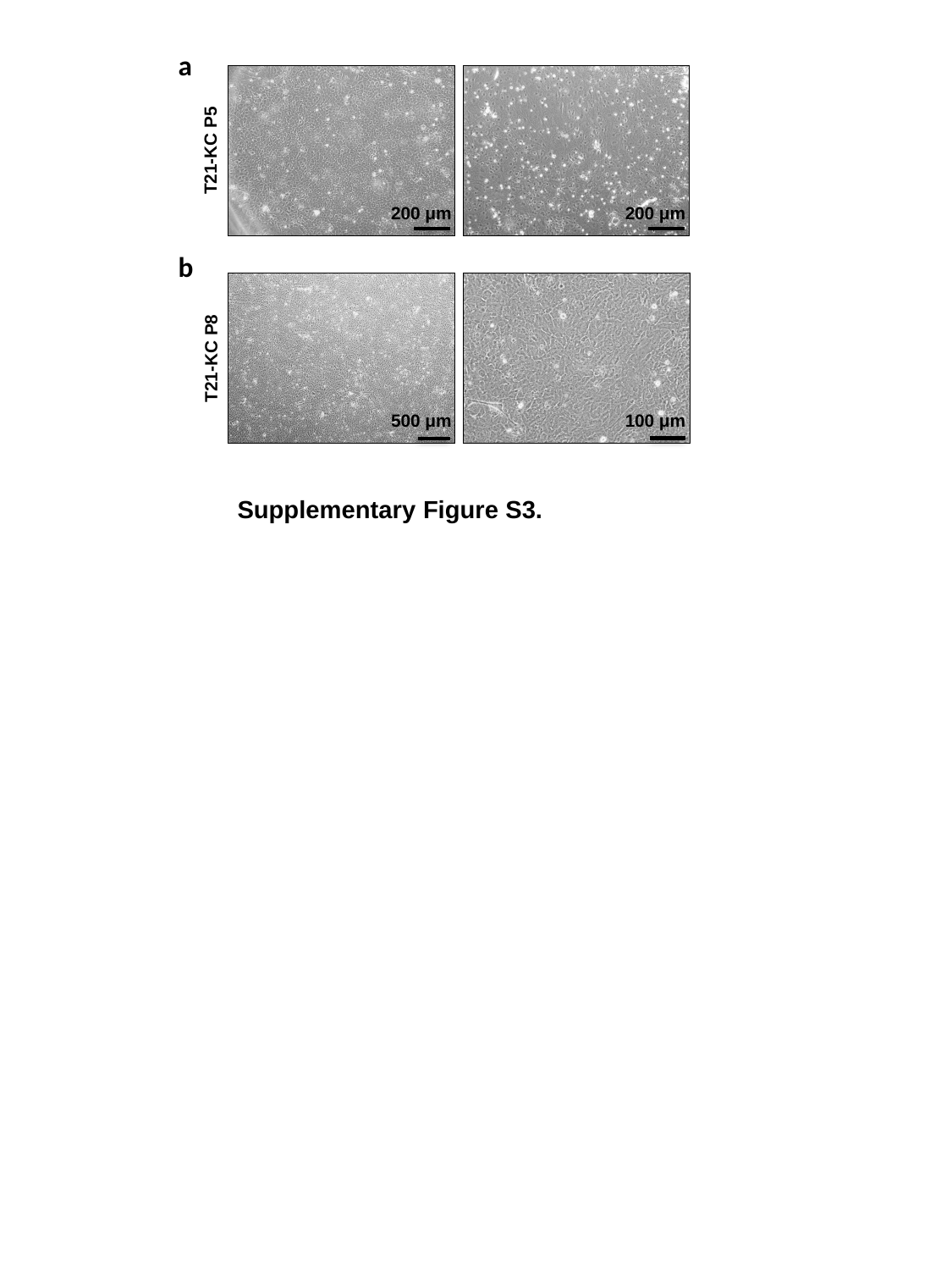

a
T21-KC P5
200 μm
200 μm
b
T21-KC P8
500 μm
100 μm
Supplementary Figure S3.

Supplement: Supplementary file 5 — Additional file 5 Figure S3. Colonial isolation of T21-KC (Related to Fig. 4). A Phase-contrast photomicrographs of T21-KC before colony isolation. B Phase-contrast photomicrographs of T21-KC after three colony isolations. After three times of isolations from passage 6 to passage 8, keratinocyte-like-cells were observed all over the dish. However, Fig. 4H showed that cytokeratins were not expressed in these cells. [file 13287_2021_2448_MOESM5_ESM.pptx]
